# Supplementary material for: Dedifferentiation-Dependent Regeneration of the Biliary Ductal Epithelium in Response to Hepatic Injury in TFF1-Deficient Mice
Source: Cells. 2025 Aug 27;14(17):1323. doi: 10.3390/cells14171323 (PMC12428306; doi:10.3390/cells14171323)
Supplement: Supplementary file 1 [file cells-14-01323-s001.zip › cells-3795398-supplementary.pdf]

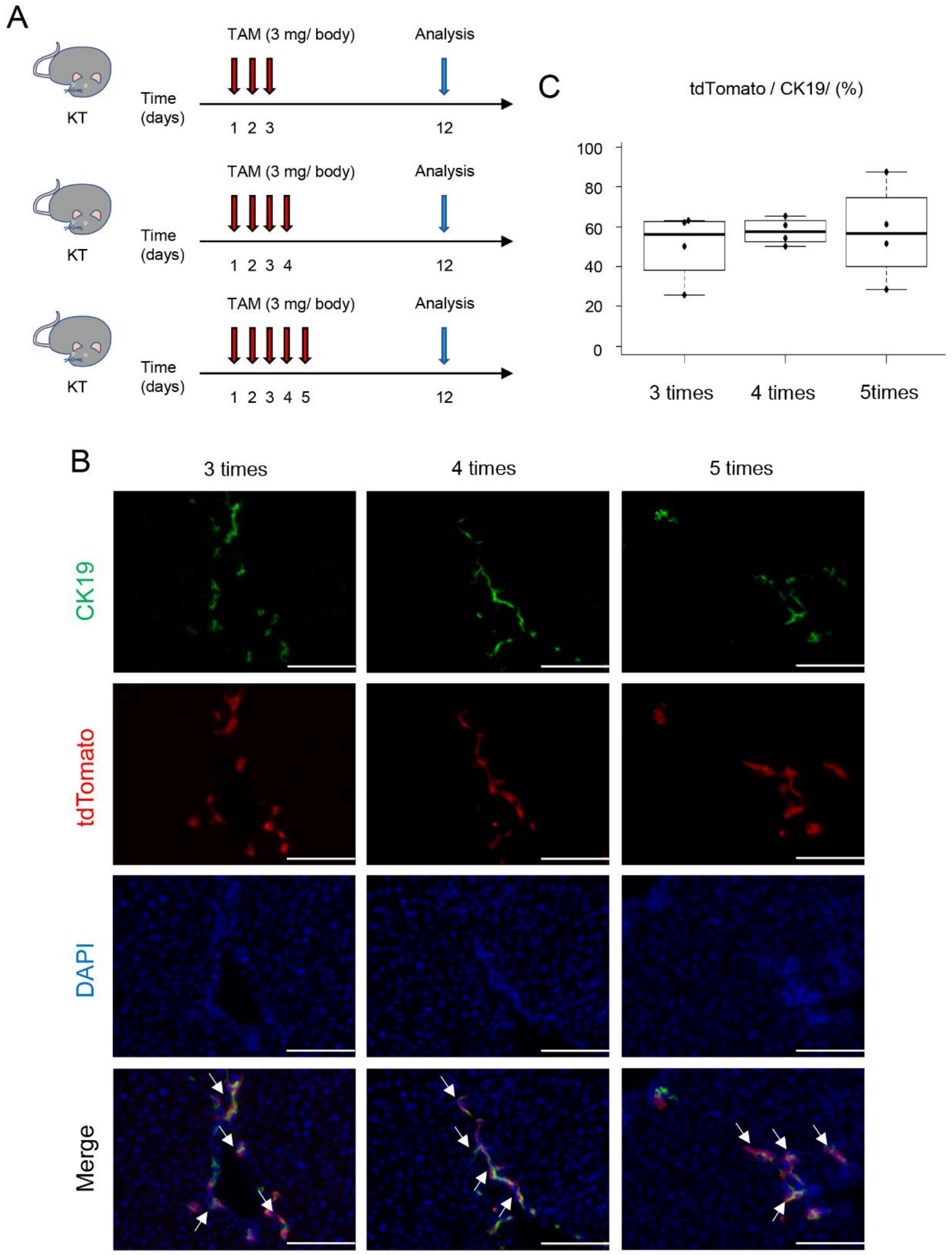

Supplemental Figure S1. Preliminary TAM administration.

(A) Scheme of mouse treatment with TAM. (B) Representative fluorescence images of CK19 and tdTomato. The arrows indicate tdTomato-labeled BECs. (C) Quantification of the tdTomato-labeled BECs in the KT (n=4), KT/TFF1$^{+/-}$ (n=4) and KT/TFF1$^{-/-}$ mice (n=4) before treatment. Scale bars: 100 μm.

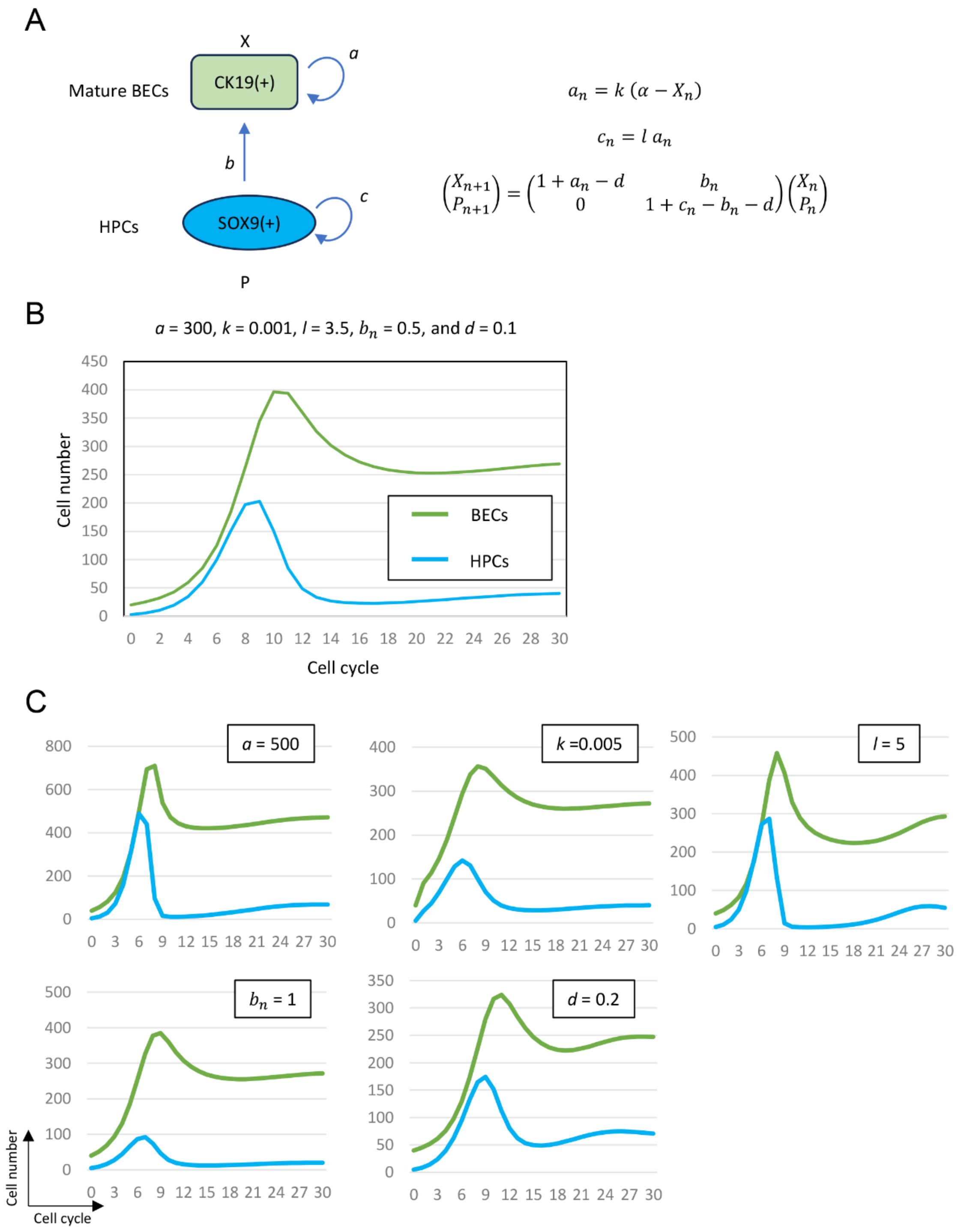

Supplemental Figure S2. Mathematical model of HPC-dependent regeneration of BECs.

(A) Scheme and equation of the mathematical model for HPC-dependent regeneration of BECs. (B) Simulation of the dynamic change in the number of BECs and HPCs. (C) Sensitivity analysis with various values of coefficient and constant.

A

	cell cycle	0	5	10	15	20	25	30	35	40
KT	BECs proliferation (a)	0.22	0.19	0.10	0.09	0.09	0.10	0.10	0.10	0.10
	differentiation (b)	0.46	0.48	0.48	0.47	0.47	0.47	0.47	0.47	0.47
	HPCs proliferation (c)	0.77	0.66	0.37	0.31	0.33	0.33	0.33	0.33	0.33
	dedifferentiation (e)	0.04	0.02	0.02	0.03	0.03	0.03	0.03	0.03	0.03
KT/TFF1+/-	BECs proliferation (a)	0.15	0.17	0.08	0.08	0.09	0.09	0.09	0.09	0.09
	differentiation (b)	0.41	0.47	0.46	0.45	0.45	0.45	0.45	0.45	0.45
	HPCs proliferation (c)	0.53	0.59	0.26	0.29	0.30	0.30	0.30	0.30	0.30
	dedifferentiation (e)	0.09	0.03	0.04	0.05	0.05	0.05	0.05	0.05	0.05
KT/TFF1-/-	BECs proliferation (a)	0.02	0.12	0.22	0.04	0.08	0.08	0.08	0.08	0.08
	differentiation (b)	0.26	0.35	0.50	0.42	0.43	0.43	0.43	0.43	0.43
	HPCs proliferation (c)	0.07	0.41	0.76	0.12	0.29	0.28	0.28	0.28	0.28
	dedifferentiation (e)	0.24	0.15	0.00	0.08	0.07	0.07	0.07	0.07	0.07

B

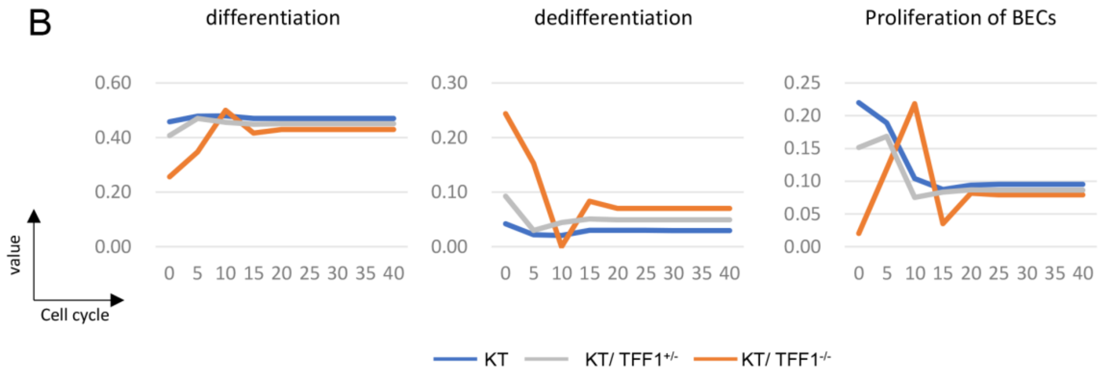

Supplemental Figure S3. Dynamic change of each coefficients.

(A) The value of each coefficients at representative cell cycles. (B) Graphs showing the dynamic change of differentiation ratio, dedifferentiation ratio, and proliferation ratio of BECs.
